# Carriage of *Haemophilus influenzae* in the Pre- and Post-Hib Vaccine Eras Revisited: A Systematic Review and Meta-Analysis

**DOI:** 10.3390/vaccines14060542

**Published:** 2026-06-20

**Authors:** Samy Taha, Nouria Belkacem, Ala-Eddine Deghmane, Muhamed-Kheir Taha

**Affiliations:** Institut Pasteur, Invasive Bacterial Infections Unit, National Reference Centre for Meningococci and Haemophilus influenzae, 75724 Paris Cedex 15, France; samy.taha@pasteur.fr (S.T.); nouriabelkacem@hotmail.fr (N.B.); ala-eddine.deghmane@pasteur.fr (A.-E.D.)

**Keywords:** *Haemophilus influnezae*, carriage, vaccination, serotype b, metanalysis, vaccination schedule

## Abstract

**Background/Objectives**: Re-emergence of *Haemophilus influenzae* serotype b (Hib) was reported in several European countries. We aimed to characterize the age distribution of *H. influnezae* carriage before and after Hib vaccination. **Methods**: We conducted a systematic review and meta-analysis to reassess *H. influenzae* carriage dynamics in the pre- and post-Hib vaccination eras, focusing on age-specific patterns in childhood. Searches were performed with no date restriction and included PubMed/MEDLINE, Scopus, Web of Science, WHO Global Index Medicus, and the Cochrane Library. Eligible studies reported nasopharyngeal and/or oropharyngeal carriage prevalence and serotype distribution. Pooled estimates with 95% confidence intervals (CIs) were calculated using random-effects models, with age-stratified analyses. **Results**: Twenty-two studies were included (12 pre- and 10 post-Hib vaccination). Pre-vaccination, pooled *H. influenzae* carriage prevalence was 24.3% (95% CI, 18.9–30.7%), including 6% (95% CI, 3.4–12.8%) for Hib and 17.5% (95% CI, 12.6–23.9%) for non–type b strains. Post-vaccination, overall carriage remained similar (21.8%; 95% CI, 14.6–31.2%), but Hib carriage declined markedly to 0.67% (95% CI, 0.26–1.71%), while non–type b strains predominated (16.7%; 95% CI, 10.4–25.6%). Meta-analysis showed that carriage peaked around 4–5 years of age and persisted into later childhood. **Conclusions**: Hib vaccination has reduced Hib carriage, but overall *H. influenzae* carriage persists due to non–type b strains. Age-related persistence of carriage may have implications for herd protection, particularly in the context of evolving vaccination schedules with early childhood boosters. Continued surveillance integrating carriage and immunological data is needed to inform optimization of vaccination strategies.

## 1. Introduction

*Haemophilus influenzae* is a frequent colonizer of the human nasopharynx and an important cause of invasive disease, particularly in young children and immunocompromised individuals, and it is dominated by bacteremia and meningitis [1]. Prior to the introduction of the *H. influenzae* type b (Hib) conjugate vaccine, serotype b was the predominant cause of bacterial meningitis and other invasive infections in children aged <5 years [2,3]. The widespread implementation of Hib vaccination in the 1990s resulted in a substantial and sustained decline in invasive disease attributable to this serotype across multiple settings [4]. However, recent works in some settings have reported a re-emergence of Hib disease occurring despite age-appropriate vaccination, as well as an increase in *H. influenzae* serotype a disease [5,6,7,8]. Data also suggest that a rapid decline in vaccine-induced IgG against polyribosylribitol phosphate (PRP) could have contributed to this re-emergence, most likely due to a suboptimal schedule with two primary doses and an early booster dose [5]. Few studies simultaneously measured carriage prevalence and invasive Hib incidence in the same population over time. However, several studies emphasized the importance of carriage in the disease control [9,10,11]. Nevertheless, nasopharyngeal carriage of *H. influenzae* persists and remains central to transmission dynamics and disease epidemiology. Asymptomatic carriage constitutes the primary reservoir for invasive disease and underpins population-level spread [12]. Several studies have identified younger age as the strongest predictor of *H. influenzae* carriage, with the highest colonization rates during early childhood that decline thereafter. Other factors associated with carriage include seasonality, household and daycare exposures, recent respiratory infections, and interactions with other nasopharyngeal bacterial species. These factors likely contribute to geographic variation in carriage prevalence and to the persistence of *H. influenzae* circulation despite widespread Hib vaccination [13]. In the post-Hib era, several studies have reported shifts in carriage and disease patterns, characterized by a relative increase in non–type b encapsulated strains and non-typeable *H. influenzae* [14]. However, the magnitude and consistency of these changes vary across regions and study populations. Systematic surveillance of *H. influenzae* carriage is therefore critical for assessing the long-term impact of Hib vaccination programs and for identifying potential gaps in herd protection [15]. Carriage data also provide an early indicator of changes in serotype distribution and allow the detection of emerging strains with increased invasive potential [16,17]. In addition, carriage studies offer insights into nasopharyngeal microbial ecology and interactions with co-colonizing respiratory pathogens, which may influence both acquisition and clearance [18]. However, no quantitative synthesis has clearly described how age-stratified carriage patterns differ between the pre- and post-Hib vaccine eras, particularly in early childhood.

In this systematic review and meta-analysis, we synthesized available data on nasopharyngeal carriage of *H. influenzae* across different geographic regions, epidemiological contexts, and age distributions. We focused on pooled carriage prevalence estimates, serotype and age distribution, and the influence of Hib vaccination status. This study aimed to quantitatively synthesize available data on *H. influenzae* carriage according to age, serotype, and vaccination context, and to assess how these patterns may help explain the re-emergence of invasive Hib disease.

## 2. Materials and Methods

### 2.1. Protocol Registration

This review was conducted and reported in accordance with the Preferred Reporting Items for Systematic Reviews and Meta-Analyses (PRISMA) guidelines [19]. The detailed protocol for this systematic review and meta-analysis was registered on 29 April 2026 and publicly available on Open Science Framework (https://osf.io/bhvc2, accessed on 16 June 2026), and the PRISMA checklist is provided as Supplementary Materials.

### 2.2. Search Strategy

A systematic literature search was conducted in PubMed/MEDLINE, Scopus, Web of Science (Core Collection), WHO Global Index Medicus (including its regional databases), and the Cochrane Central Register of Controlled Trials (CENTRAL), from database inception to 3 August 2025. There were no restrictions on language or publication dates. Exploratory searches were performed in Google Scholar as a supplementary source for exploratory checking of potentially missed references, but records identified there were not included in the final dataset because of platform-related export limitations. No formal gray literature search was conducted. While this may have resulted in some unpublished studies not being identified, the review included comprehensive searches of major international bibliographic databases, and the primary outcomes required detailed microbiological and epidemiological reporting that is typically available only in peer-reviewed publications. Potentially unique references were captured through the WHO Global Index Medicus and the Cochrane Library. Search strategies combined terms related to *Haemophilus influenzae*, nasopharyngeal carriage or colonization, anatomical site, and serotype. Database-specific syntax was adapted as required. The full search strategies are provided on (https://osf.io/bhvc2 (accessed on 16 June 2026)) and in Appendix A.

### 2.3. Study Selection

Studies were eligible if they reported original data on nasopharyngeal carriage of *Haemophilus influenzae*, including serotype distribution (capsulated and/or non-typeable strains), and a clearly defined study population and study period. Studies reporting nasopharyngeal and/or oropharyngeal carriage were included and jointly analyzed as pharyngeal carriage. We excluded studies limited to invasive disease isolates, outbreak investigations, case reports, reviews, or editorials, and studies using non-nasopharyngeal specimens only. All retrieved records were merged into a single master database and deduplicated using a stepwise procedure based on exact matching of DOI and PMID, followed by normalized title matching and fuzzy title similarity. An initial automated pre-screening was then applied to exclude records that clearly did not mention at least one of the following elements in the title or abstract: *H. influenzae* carriage, serotype, study period, or population. The remaining records (*n* = 493) underwent title and abstract screening independently by all four authors. Studies were retained when at least three of the four reviewers judged them potentially eligible and were excluded when none or only one reviewer supported inclusion. When two reviewers supported inclusion, and two did not, final arbitration was performed by the senior author (MKT). Full-text assessment was then performed for all studies retained after this consensus-based screening process.

### 2.4. Data Extraction

Data were extracted independently by at least two reviewers using a standardized extraction form. Each study’s data were extracted for the whole study population and stratified according to the available variables. Variables included study design, country and region, study period, population characteristics, laboratory methods, vaccination context, overall carriage prevalence, and serotype-specific carriage prevalence.

### 2.5. Study Quality Assessment

Study quality was assessed independently by two reviewers (ST and MKT), and risk of bias was assessed using the Joanna Briggs Institute (JBI) Critical Appraisal Checklist for Studies Reporting Prevalence Data [20]. Studies were evaluated across domains related to sampling, measurement, and analysis, with disagreements resolved by consensus. Sources of between-study heterogeneity, including a detailed age group distribution for <5 years of age, socioeconomic setting, study period, and methodological differences, were explored. Inter-reviewer agreement was also assessed using Fleiss’ kappa statistic, a chance-corrected measure of agreement among multiple raters [21]. Agreement was calculated from the independent evaluation of the 493 studies selected for detailed screening by four reviewers.

### 2.6. Classification of Studies by Vaccination Era

Included studies were classified according to the timing of Hib conjugate vaccine implementation extracted in the study setting. The date of this implementation differed among countries. Studies conducted entirely before routine Hib vaccine introduction were categorized as pre-Hib, while studies conducted after national or regional implementation of Hib vaccination programs were categorized as post-Hib. For studies spanning the year of Hib implementation, classification was based on whether most of the sample collection occurred before or after implementation; when this could not be determined reliably, studies were classified using national program timelines. Pre- and post-Hib studies were analyzed separately to allow comparison of carriage patterns before and after vaccine implementation.

### 2.7. Age Group Harmonization and Age Mapping

Reported age categories varied substantially across studies, ranging from narrow strata (e.g., 12–23 months) to broad age ranges (e.g., <5 years or ≥10 years). To enable age-based analyses, all age strata were standardized using a structured age-mapping procedure. For each reported age stratum, minimum and maximum ages were extracted and converted to years. When ages were reported in months, values were converted to years prior to analysis. An age-band midpoint (in years) was then calculated for each stratum and used as a continuous age variable in meta-regression analyses. In parallel, age strata were classified into predefined strict age bands (<1 year, 1–4 years, 5–9 years, ≥10 years). Strata spanning multiple bands were labeled as *mixed* and excluded from strict age-band analyses but retained for midpoint-based analyses. This dual approach allowed the inclusion of the maximum number of data points while preserving biologically interpretable age categories.

### 2.8. Outcome Definitions

Carriage outcomes were analyzed separately for Hib (serotype b) and non–type b *H. influenzae*, including non-typeable strains and other encapsulated serotypes, as many studies did not report non-typeable and non-b encapsulated strains separately. For each stratum, prevalence was calculated as the number of carriers divided by the population sampled.

### 2.9. Data Synthesis and Statistical Analysis

Pooled prevalence estimates and corresponding 95% confidence intervals were calculated using a random-effects meta-analysis. Between-study heterogeneity was assessed using the I^2^ statistic. Subgroup and sensitivity analyses explored sources of heterogeneity, including age group, geographic region, and vaccination context. Missingness was addressed at the study-selection stage rather than during statistical analysis. Publications that did not provide sufficient information on age distribution and/or serotype-specific carriage prevalence were excluded from quantitative synthesis. Consequently, no imputation of missing values was performed, and all analyses were based on complete available data from included studies.

#### 2.9.1. Meta-Analysis and Forest Plot

Forest plots were generated separately for pre-Hib and post-Hib periods and for Hib and non–type b outcomes. Both stratum-level and study-level visualizations were explored; study-level estimates were prioritized for pooled analyses to avoid over-weighting studies reporting multiple age strata.

#### 2.9.2. Age–Prevalence Meta-Regression

To explore age-related trends in carriage prevalence, random-effects meta-regression models were fitted using age-band midpoints as a continuous moderator. Models were fitted separately for Hib and non–type b outcomes and for pre- and post-Hib periods. Predicted prevalence curves with 95% confidence intervals were generated across the observed age range. In addition, 95% prediction intervals were calculated to reflect between-study heterogeneity. Observed study estimates were overlaid on these curves, with marker size proportional to study sample size.

#### 2.9.3. Funnel Plots and Assessment of Small-Study Effects

Potential small-study effects were explored using funnel plots constructed at the study level. Egger’s regression test was performed when the number of studies was sufficient.

#### 2.9.4. Software

All analyses were performed using Python 3.9.11 with Pandas version 2.3.1, NumPy version 2.0.2, SciPy version 1.13.1, Statsmodels version 0.14.5, and Matplotlib version 3.9.4.

## 3. Results

### 3.1. Study Selection and Characteristics

**Figure 1:**
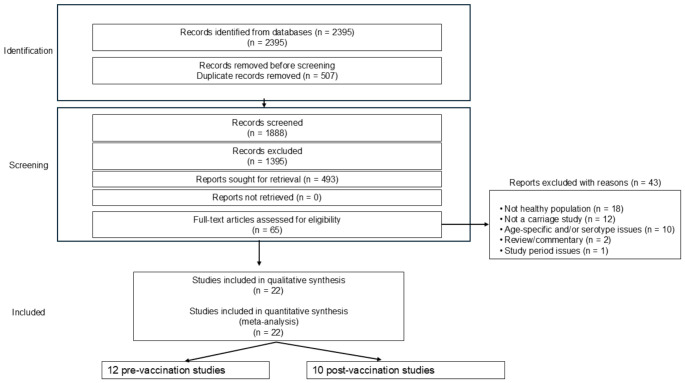
PRISMA flow diagram of study selection. PRISMA flow diagram showing study identification, screening, eligibility, and inclusion in the systematic review and meta-analysis of nasopharyngeal carriage of *H. influenzae*.

The systematic literature search and successive screening steps resulted in the inclusion of 22 studies in the meta-analysis and were included in the meta-analysis, comprising 12 studies conducted before Hib vaccine implementation (pre-Hib) [22,23,24,25,26,27,28,29,30,31,32,33] and 10 studies conducted after Hib vaccine implementation (post-Hib) [10,34,35,36,37,38,39,40,41,42] (Figure 1 and Supplementary Table S1).

Two reports from Kenya [42,43], conducted during distinct post-Hib periods, were included as separate study contributions after verification that their sampled populations and sample sizes did not overlap. Included studies were geographically diverse and reported nasopharyngeal carriage of *H. influenzae* across a wide range of age groups. Age stratification varied substantially between studies, with reported age categories ranging from narrow strata (e.g., 12–23 months) to broad groupings (e.g., <5 years or ≥10 years). Following age harmonization, all studies contributed at least one age stratum to midpoint-based analyses, while a subset contributed data to strict age-band analyses (<1 year, 1–4 years, 5–9 years, ≥10 years).

### 3.2. Pre-Hib Vaccination Carriage of Haemophilus influenzae

In the pre-Hib period, Hi carriage rates varied across studies and age groups. The overall pooled carriage rate of Hi was 24.3% (95% CI 18.9–30.7, I^2^ = 96.4%). Sensitivity analysis for the geographic region (after excluding Turkish studies) showed similar results Supplementary Figure S1.

### 3.3. Hib Carriage Before Hib Vaccination

**Figure 2:**
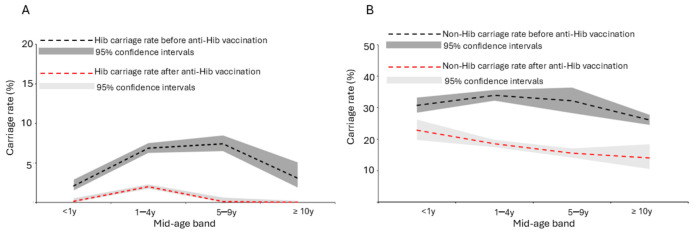
Carriage rate per group of mid-age using bin-wise weighted means connected by lines. Overall observed prevalence within each age band (<1”, “1–4”, “5–9”, “≥10”), weighted by sample size. (**A**) Hib carriage rates for the periods pre- and post-Hib. (**B**) Non–type b carriage rates for the period pre- and post-Hib.

**Figure 3:**
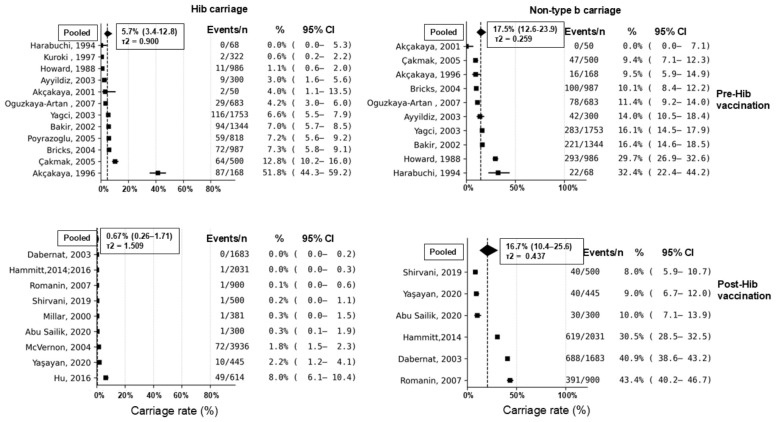
Forest plot describing the carriage rate of Hib and other Hi isolates before and after Hib vaccination. Studies were grouped per vaccination period and per type of isolates (Hib and non–type b, including NT and other capsulated Hi). Pooled estimations are shown within boxes. For each study, Events/*n* indicate the number of carriers (Events) on the total number of subjects (*n*). The carriage percentage per study and it 95% CI are also shown. Black symbols indicate pooled random-effects estimates with their 95% confidence intervals. The studies shown in this forest plot correspond to the included studies [10,22,23,24,25,26,27,28,29,30,31,32,33,34,35,36,37,38,39,40,41,42,43].

Hib carriage accounted for a small proportion even before Hib vaccination, as depicted in bin-wise means of the data that also showed age variation with a peak that seems to occur at 4–5 years of age (Figure 2). Exploratory age–prevalence meta-regression suggested an age-related pattern, with higher Hib carriage observed among children aged 1–4 years and 5–9 years of age and declining prevalence with increasing age (Supplementary Figure S2). Forest plot analyses for Hib carriage before vaccination revealed a pooled Hib carriage rate was 6.7% (95% CI 3.4–12.8) (Figure 3).

**Figure 4:**
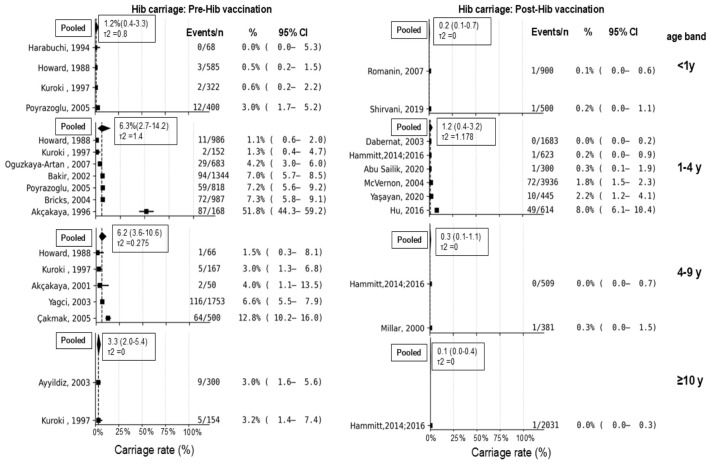
Forest plot describing the carriage rates of Hib. *H. influenzae* Hib isolates before and after Hib vaccination. Subgroup analyses by mid-age groups. Pooled estimations are shown within boxes. For each study, Events/*n* indicate the number of carriers (Events) on the total number of subjects (*n*). The carriage percentage per study and it 95% CI are also shown. Black symbols indicate pooled random-effects estimates with their 95% confidence intervals. The studies shown in this forest plot correspond to the included studies [10,22,23,24,25,26,27,28,29,30,31,32,33,34,35,36,37,38,39,40,41,42,43].

Forest plot analyses per age band midpoints shown in Figure 4 suggest between-study heterogeneity in the pre-Hib vaccination era, but this varied across age groups, with the highest heterogeneity for the age midpoint of 1–4 years of age (τ^2^ = 1.4). Pooled random-effects estimates indicated non-negligible Hib carriage, particularly in the age groups 1–4 years and 5–9 years (pooled carriage rates were 6.3% and 6.2%, respectively), but it was lower among children aged <1 year of age (pooled carriage rate 1.2%).

Non–type b *H. influenzae* carriage was consistently more prevalent than Hib carriage in the pre-Hib period. Age–prevalence curves suggested increasing non–type b carriage with age, with the highest prevalence observed in older children and adults (Figure S2 and Figure 3). Forest plot analyses for non–type b carriage before vaccination revealed a pooled non–type b carriage rate of 17.5% (pooled carriage rate 17.5%, 95% CI 12.6–23.9), reflecting moderate heterogeneity across studies for non–type B carriage (Figure 4).

### 3.4. Post-Hib Vaccination Carriage of H. influenzae

In the post-Hib vaccination period, overall *H. influenzae* carriage decreased slightly but remained common, with a pooled prevalence of 21.8% (95% CI 14.6–31.2; I^2^ = 98.7%), broadly similar to pre-Hib estimates despite wide heterogeneity.

### 3.5. Hib Carriage After Hib Vaccination

Following Hib vaccine implementation, Hib carriage prevalence was markedly reduced across most studies. Bin-wise means of the data showed a significant reduction in the age-suggested peak at 4–5 years of age with non-overlapping 95% CI, with the almost complete disappearance of carriage among the group of 5–9 years of age (Supplementary Figure S2). Age–rate meta-regression showed consistently low predicted Hib carriage across all age groups, with prediction intervals indicating limited variability between studies (Supplementary Figure S3). Many post-Hib studies reported zero or near-zero Hib carriage, particularly in vaccinated pediatric populations.

The pooled overall Hib carriage rate was 0.67% (95% CI 0.3–1.6) (Figure 3), with a low level of carriage that persisted in the group of 1–4 years of age and declining prevalence with increasing age (Figure 2).

Forest plot analyses per age band midpoints shown in Figure 4 confirmed lower Hib carriage estimates across all analyzed age groups in the post-Hib period and, in particular, the group of 5–9 years of age, although some degree of heterogeneity persisted, particularly in the group of 1–4 years of age (τ^2^ = 1.4).

### 3.6. Non–Type b Carriage

In contrast, non–type b *H. influenzae* carriage remained common in the post-Hib period (Figure 2 and Figure S2). Pooled estimates were similar to those observed in the pre-Hib period, 16.7% (95% CI 10.4–25.6).

**Figure 5:**
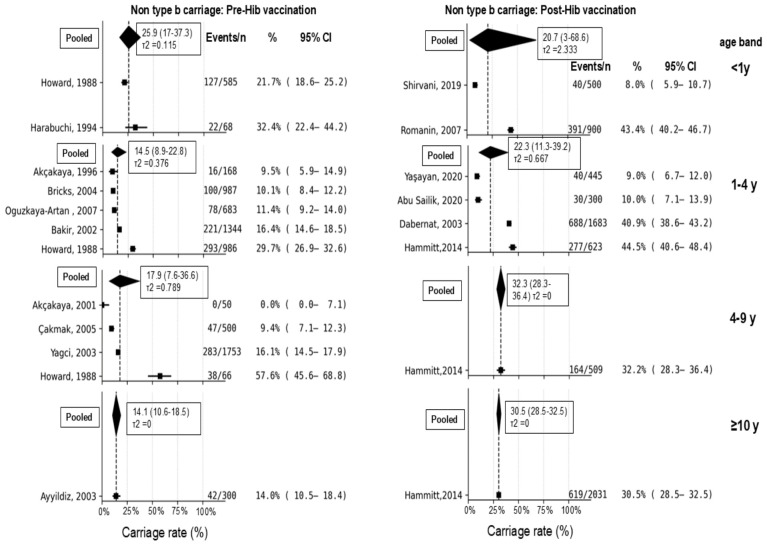
Forest plot describing the carriage rates of non–type b. Non–type b isolates before and after Hib vaccination. Non–type b includes NT and other capsulated Hi. Subgroup analyses by mid-age groups. Pooled estimations are shown within boxes. For each study, Events/n indicate the number of carriers (Events) on the total number of subjects (n). The carriage percentage per study and it 95% CI are also shown. Black symbols indicate pooled random-effects estimates with their 95% confidence intervals. The studies shown in this forest plot correspond to the included studies [22,23,24,25,26,27,28,29,30,31,32,33,34,35,36,37,38,39,40,41].

Age–prevalence curves indicated sustained or increasing carriage with age, with the highest prevalence observed in older age groups (Figure 5). However, prediction intervals remained wide, and the high τ^2^ values suggested substantial heterogeneity between studies (Figure 2 and Figure 5).

All data from the pre- and post-Hib analyses revealed a pronounced reduction in Hib carriage following vaccine implementation, consistent across age groups. In contrast, non–type b carriage showed no corresponding decline and remained the dominant contributor to overall *H. influenzae* carriage in the post-Hib era.

### 3.7. Funnel Plot Analyses and Small-Study Effects

Funnel plots constructed at the study level for pre- and post-Hib periods are shown in Supplementary Figure S3. Funnel plots for pre-Hib carriage similarly demonstrated variability among smaller studies, with no consistent pattern suggestive of selective reporting (Egger intercept −2.995, *p* = 0.005 but the intercept was −4.321, *p* = 0.051 in sensitive analysis excluding small studies (Supplementary Figure S3E). Pre-Hib non–type b *H. influenzae* carriage showed a broadly symmetrical distribution around the pooled effect estimate, with some dispersion among smaller studies, consistent with between-study heterogeneity rather than clear evidence of publication bias (Egger intercept −3.317, *p* = 0.003) (Supplementary Figure S3B).

In contrast, funnel plots for post-Hib analyses were based on a small number of studies (<10), which did not allow Egger test evaluation and were dominated by sparse data and zero or near-zero Hib carriage estimates. As a result, funnel plot shapes in the post-Hib period were poorly defined and not considered informative for the assessment of publication bias (Supplementary Figure S3C,D). However, the observed asymmetry is likely to reflect true between-study heterogeneity and era-related differences rather than selective publication.

### 3.8. Risk of Bias Assessment

Risk-of-bias assessment using the Joanna Briggs Institute (JBI) checklist indicated generally significant methodological quality across the included studies. Most studies satisfied the majority of appraisal criteria, particularly those relating to sample selection, measurement of carriage outcomes, and statistical reporting. Areas of uncertainty were primarily related to incomplete reporting of response rates and sampling procedures. Overall, the risk of bias was considered low to moderate, supporting the robustness of the pooled estimates. Moreover, independent assessment of the 493 studies demonstrated almost perfect agreement among reviewers (Fleiss’ κ = 0.82), with an observed agreement of 96.6%. Discrepancies were resolved through discussion and consensus.

## 4. Discussion

Across 22 studies spanning multiple geographic regions, a marked reduction in Hib carriage was observed following vaccine introduction, accompanied by persistent and age-dependent carriage of non–type b *H. influenzae*. The use of age-band midpoints allowed the exploration of age-related trends despite substantial heterogeneity in reported age strata across studies. Exploratory age-pattern analyses suggested that pre-Hib carriage was highest in childhood, possibly peaking at 4–5 years of age and declining thereafter, whereas non–type b carriage increased with age in both pre- and post-Hib vaccination periods. The inter-studies variability likely reflects differences in study design, sampling methods, geographic settings, vaccination coverage, and circulating *H. influenzae* populations.

Model-based age–prevalence curves were consistent with bin-wise weighted prevalence estimates across age groups, with both approaches demonstrating similar patterns of carriage by age. In many post-Hib studies, Hib carriage was absent or detected at very low levels, particularly among vaccinated pediatric populations. These results are consistent with the established effects of Hib conjugate vaccines in reducing nasopharyngeal carriage and interrupting transmission, thereby contributing to both direct and indirect (herd) protection [44]. The meta-analysis suggests that the reduction in carriage was remarkable around the peak of pre-Hib vaccine carriage (4–5 years of age). However, these meta-regressions should be interpreted as exploratory because age strata from the same study could contribute more than once, and within-study correlation was not explicitly modeled. Protection against acquisition of carriage and colonization requires higher levels of anti-PRP antibodies than the threshold of long-term protection against invasive infection (≥5 µg/mL versus 1 µg/mL, respectively) [45]. Achieving high levels of anti-Hib antibodies at this age may be crucial to maintain herd protection in other unvaccinated or partially vaccinated population groups. Such high levels may be achieved after a booster dose during the second year of life, a scheme that is used in most countries from which studies were included. The French context is of particular interest. The French pre-Hib-era carriage study included in this meta-analysis was performed under the former 3 + 1 schedule (2, 3, 4 months with a booster between 16 and 18 months of age) [40], whereas the recent increase in invasive Hib disease was observed after adoption of a 2 + 1 schedule with an earlier booster (2 and 4 months with a booster at 11 months of age) [5]. This interpretation remains indirect because contemporary carriage data are lacking, and dedicated carriage studies are needed to evaluate this hypothesis directly. Together with seroprevalence data, our findings raise the possibility that lower anti-PRP IgG levels after the earlier booster schedule could contribute to less sustained suppression of Hib circulation in later childhood [5]. Together with seroprevalence data, our findings raise the hypothesis that this reemergence may be due to lower levels of anti-PRP IgG after the early booster dose at 11 months of age [5]. This hypothesis raises the possibility that lower antibody levels may be insufficient to fully prevent Hib circulation in later childhood. Consistent with these findings, an increase in Hib disease was detected among children aged 4 years in France in 2018 [46,47]. This circulation may then be responsible for transmission of Hib isolates to incompletely vaccinated infants [46] and potentially to adults. However, this interpretation remains indirect, and a new carriage study may be warranted in France to explore this hypothesis. A booster dose at the age of 4–5 years can be one measure to implement. The persistence of low-level Hib carriage observed in our analysis supports the need for integrated surveillance combining carriage studies, serological monitoring, and invasive disease surveillance to better understand the mechanisms underlying occasional re-emergence of Hib disease in highly vaccinated populations [11,48]. Several children’s groups have consistently been identified as having lower or less persistent anti-PRP antibody concentrations, and these populations are considered at increased risk of Hib colonization and invasive disease [49].

In contrast to Hib, non–type b *H. influenzae* carriage remained common in both pre- and post-Hib periods. Age–prevalence analyses consistently showed increasing carriage with age, with the highest prevalence observed in older children and adults. Importantly, no reduction in non–type B carriage was observed following Hib vaccine implementation. This observation may be due to the high carriage rate of non-typeable isolates that was observed in our analysis (Figure 3).

Substantial heterogeneity was observed in both pre- and post-Hib analyses, reflecting the diversity of included studies and the ecological nature of carriage data. Funnel plot analyses did not reveal strong evidence of small-study effects, although interpretation was limited by the number of studies and the presence of zero-event strata, particularly for Hib in the post-Hib period.

Several limitations should be considered when interpreting these findings. First, the included studies were predominantly cross-sectional carriage surveys, with only a small number employing longitudinal follow-up, limiting the ability to assess temporal changes in individual carriage status or causal relationships between risk factors and carriage. Second, although study quality was systematically assessed using a standardized risk-of-bias tool and most studies were judged to be of moderate to high methodological quality, differences in sampling strategies, age-group definitions, laboratory methods, and reporting practices contributed to substantial between-study heterogeneity. Finally, the external validity of the findings may be limited, as a considerable proportion of the included studies originated from a small number of countries, particularly Turkey, and may not fully represent carriage dynamics in regions with different epidemiological, socioeconomic, or vaccination contexts. Therefore, caution is warranted when generalizing the pooled estimates to all populations and settings.

## 5. Conclusions

This meta-analysis suggests that the greatest reduction in carriage occurred in the age range where pre-Hib carriage appeared highest. These findings support the hypothesis that maintaining sufficiently high anti-PRP antibody levels beyond infancy may contribute to sustained control of Hib carriage and herd protection.

## Data Availability

All data supporting the findings of this study are available within the paper and its Supplementary Information.

## Supplementary Materials

The following supporting information can be downloaded at: https://www.mdpi.com/article/10.3390/vaccines14060542/s1. Figure S1: Forest plot describing the carriage rate of Hi isolates. Figure S2: Age–versus carriage meta-regression analyses; Figure S3: Funnel plot analysis; Table S1: Included studies and their characteristics, and Supplementary Material 1: the PRISMA checklist.

## Abbreviations

The following abbreviations are used in this manuscript:

Hib	*Haemophilus influnezae* serotype b.
Non-Hib	*Haemophilus influnezae* of other serotypes or non-typeable.

## Appendix A

**Search strategy**

Full search strategies by database.

The full electronic search strategies were developed using a harmonized conceptual framework combining terms related to *Haemophilus influenzae*, carriage or colonization, anatomical site, and serotype. Database-specific syntax was adapted as required.

**PubMed/MEDLINE**

(“*Haemophilus influenzae*”[Mesh] OR “*Haemophilus influenzae*”[tiab]).

AND (carriage[tiab] OR coloniz*[tiab] OR “carrier state”[Mesh]).

AND (nasopharyn*[tiab] OR oropharyn*[tiab]).

AND (serotype OR Hib OR non-typeable OR NTHi).

**Scopus**

TITLE-ABS-KEY(“*Haemophilus influenzae*”).

AND (carriage OR colonization OR colonization OR nasopharyn* OR oropharyn*).

AND (serotype OR Hib OR NTHi).

**Web of Science (Core Collection)**

(“*Haemophilus influenzae*” AND (carriage OR colonization OR colonization).

AND (nasopharyngeal OR oropharyngeal)).

OR.

(“*Haemophilus influenzae*” AND serotype.

AND (carriage OR colonization OR colonization)).

**WHO Global Index Medicus**

Adapted versions of the above search strategies were applied across the regional databases of WHO Global Index Medicus (AIM, LILACS, IMSEAR, WPRIM, IMEMR).

**Cochrane Library (CENTRAL)**

Adapted versions of the above search strategies were applied to the Cochrane Central Register of Controlled Trials (CENTRAL).
