# Supplementary material for: Carriage of Haemophilus influenzae in the Pre- and Post-Hib Vaccine Eras Revisited: A Systematic Review and Meta-Analysis
Source: Vaccines (Basel). 2026 Jun 20;14(6):542. doi: 10.3390/vaccines14060542 (PMC13308107; doi:10.3390/vaccines14060542)
Supplement: Supplementary file 1 [file vaccines-14-00542-s001.zip › Excluded_References_N43.pdf]

| Title | Year | DOI | PMID |
|---|---|---|---|
| Decreased prevalence of Moraxella catarrhalis in addition to Streptococcus pneumoniae in children with upper respiratory tract infection after introduction of conjugated pneumococcal vaccine: a | 2021 | 10.1016/j.cmi.2020.04.033 | 32360778 |
| Pneumococcal carriage in children attending a hospital outpatient clinic in the era of pneumococcal | 2012 | 10.1016/j.diagmicrobio.2012.07.005 | 22921813 |
| Trend change of nasopharyngeal colonization with Streptococcus pneumoniae and non-typeable Haemophilus influenzae in children attending daycare centres: nationwide population-based study, | 2021 | 10.1016/j.ijid.2021.08.065 | 34508859 |
| Incidence of Radiologically-Confirmed Pneumonia and Haemophilus influenzae Type b Carriage before Haemophilus influenzae Type b Conjugate Vaccine Introduction in Central Vietnam | 2013 | 10.1016/j.jpeds.2013.03.029 | 23773592 |
| Turnover of Haemophilus influenzae isolates in otitis-prone children | 2000 | 10.1016/S0165-5876(00)00321-9 | 10960690 |
| Epidemiology of nasopharyngeal flora in pediatric acute otitis media, from December 2000 to March | 2003 | 10.1016/S0399-077X(03)00002-7 | |
| Bacterial epidemiology of nasopharyngeal flora in children with acute otitis media | 1996 | 10.1016/S0399-077X(96)80217-4 | |
| Prevalence of Haemophilus influenzae in the nasopharynx of children from regions with varying incidence of invasive H. influenzae serotype a disease: Canadian Immunization Research Network | 2024 | 10.1080/22423982.2024.2371111 | 38941555 |
| Increasing Prevalence of Group III Penicillin-Binding Protein 3 Mutations Conferring High-Level Resistance to Beta-Lactams Among Nontypeable Haemophilus influenzae Isolates from Children in | 2019 | 10.1089/mdr.2018.0342 | 30484742 |
| THE IMPACT OF CONJUGATE VACCINE ON CARRIAGE OF HAEMOPHILUS-INFLUENZAE TYPE-B | 1995 | 10.1093/infdis/171.1.93 | 7798687 |
| Dynamics of Pneumococcal Carriage in Adults: A New Look at an Old Paradigm | 2021 | 10.1093/infdis/jiaa558 | 32877517 |
| Epidemiological Markers for Interactions Among Streptococcus pneumoniae, Haemophilus influenzae, and Staphylococcus aureus in Upper Respiratory Tract Carriage | 2016 | 10.1093/infdis/jiv761 | 26704617 |
| Streptococcus pneumoniae and noncapsular Haemophilus influenzae nasal carriage and hand contamination in children -: A comparison of two populations at risk of otitis media | 2005 | 10.1097/01.inf.0000160945.87356.ca | 15876941 |
| Impact of Haemophilus influenzae Type B Conjugate Vaccines on Nasopharyngeal Carriage in HIV-infected Children and Their Parents From West Bengal, India | 2016 | 10.1097/INF.0000000000001266 | 27753766 |
| Characteristics of Haemophilus influenzae carriage among healthy children in China: A meta- | 2023 | 10.1097/MD.0000000000035313 | 37933036 |
| DIFFERENCES IN SUBTYPE DISTRIBUTION OF HAEMOPHILUS-INFLUENZAE TYPE-B FROM CARRIERS IN THE GENERAL-POPULATION AND PATIENTS WITH MENINGITIS | 1991 | 10.1099/00222615-34-6-313 | 2056515 |
| [Nasopharyngeal Hib Carriage Among Healthy Children Attending Daycare Centers in Yokohama After One Year of a Publicly Funded Vaccine Program] | 2015 | 10.11150/kansenshogakuzasshi.89.30 | 26548294 |
| INVITRO ACTIVITY OF ORALLY-ADMINISTERED ANTIMICROBIAL AGENTS AGAINST HAEMOPHILUS-INFLUENZAE RECOVERED FROM CHILDREN MONITORED LONGITUDINALLY IN A GROUP DAY-CARE- | 1991 | 10.1128/AAC.35.10.1960 | 1759814 |
| Pharyngeal colonization dynamics of Haemophilus influenzae and Haemophilus haemolyticus in | 2007 | 10.1128/JCM.00492-07 | 17687018 |

| | | | |
|---|---|---|---|
| TURNOVER OF NONTYPABLE HEMOPHILUS-INFLUENZAE IN THE NASOPHARYNGES OF HEALTHY- | 1989 | 10.1128/JCM.27.10.2175-2179.1989 | 2584370 |
| TURNOVER OF NONENCAPSULATED HAEMOPHILUS-INFLUENZAE IN THE NASOPHARYNGES OF | 1995 | 10.1128/JCM.33.8.2027-2031.1995 | 7559942 |
| Effect of Haemophilus influenzae Type b Vaccination on Nasopharyngeal Carriage Rate in Children, | 2021 | 10.1155/2021/4923852 | 33816612 |
| Serotype Distribution and Antimicrobial Resistance Profile of Haemophilus influenzae Isolated from School Children with Acute Otitis Media | 2022 | 10.1155/2022/5391291 | 35655653 |
| The pharyngeal carriage of Haemophilus influenzae among healthy population in China: a | 2019 | 10.1186/s12879-019-4195-9 | 31226950 |
| High bacterial and viral load in the upper respiratory tract of children in the Democratic Republic of | 2020 | 10.1371/journal.pone.0240922 | 33119683 |
| Naturally acquired immunity to Haemophilus influenzae type b in healthy Cuban children | 2004 | 10.1590/S0074-02762004000700004 | 15654422 |
| MICRO-ECOLOGY OF THE NASOPHARYNGEAL BACTERIAL-FLORA IN OTITIS-PRONE AND NON- | 1993 | 10.3109/00016489309135772 | 8442428 |
| Molecular Epidemiology and Antimicrobial Resistance of Haemophilus influenzae in Adult Patients | 2020 | 10.3389/fpubh.2020.00095 | 32292774 |
| THE BACTERIOLOGY OF ACUTE PNEUMONIA AND MENINGITIS IN CHILDREN IN PAPUA-NEW-GUINEA - ASSUMPTIONS, FACTS AND TECHNICAL STRATEGIES | 1991 | | 1750263 |
| [Study of genetic diversity of nontypeable Haemophilus influenzae strains isolated from healthy children and patients with infection symptoms] | 2007 | | 17929409 |
| Low rate of nasopharyngeal carriage and high rate of ampicillin resistance for Haemophilus influenzae among healthy children younger than 5 years old in northern Taiwan | 2008 | | 18327424 |
| Multi-center surveillance for pneumonia & meningitis among children (<2 yr) for Hib vaccine probe | 2010 | | 20516536 |
| Characterization of nasopharyngeal isolates of type b Haemophilus influenzae from Delhi | 2012 | | 23287135 |
| Nasopharyngeal bacterial flora in healthy preschool children during winter-spring months | 2013 | | 23940983 |
| Effect of Biannual Azithromycin to Children under 5 Years on the Carriage of Respiratory Pathogens | 2023 | 10.4269/ajtmh.22-0583 | 36535258 |
| NASAL CARRIAGE OF PATHOGENIC BACTERIA IN KALAUNA-VILLAGE, GOODENOUGH ISLAND | 1981 | | 6978581 |
| Epidemiology of nasopharyngeal colonization with nontypeable Haemophilus influenzae in the first | 1996 | | 9082757 |
| [Etiology of acute lower respiratory tract infections among children younger than 5 years old in Santa | 1997 | | 9532829 |
| Antibiotic susceptibility patterns in H. influenzae type B isolated from healthy children oropharynx in | 2007 | | 20083276971 |
| Changing epidemiology of invasive Haemophilus influenzae in Ontario, Canada: Evidence for herd effects and strain replacement due to Hib vaccination | 2010 | 10.1016/j.vaccine.2010.03.075 | 20398617 |
| Efficacy of Haemophilus influenzae type b vaccination of children:: a meta-analysis | 2006 | 10.1007/s10096-006-0092-4 | 16491301 |
| Changes in serotype distribution of Haemophilus influenzae meningitis isolates identified through laboratory-based surveillance following routine childhood vaccination against H. influenzae type b in | 2011 | 10.1016/j.vaccine.2011.09.053 | 21945960 |
| Fall in Haemophilus influenzae serotype b (Hib) disease following implementation of a booster | 2008 | 10.1136/adc.2007.126888 | 17942585 |
