# Supplementary material for: Carriage of Haemophilus influenzae in the Pre- and Post-Hib Vaccine Eras Revisited: A Systematic Review and Meta-Analysis
Source: Vaccines (Basel). 2026 Jun 20;14(6):542. doi: 10.3390/vaccines14060542 (PMC13308107; doi:10.3390/vaccines14060542)
Supplement: Supplementary file 1 [file vaccines-14-00542-s001.zip › Supplementary Figure S1.pdf]

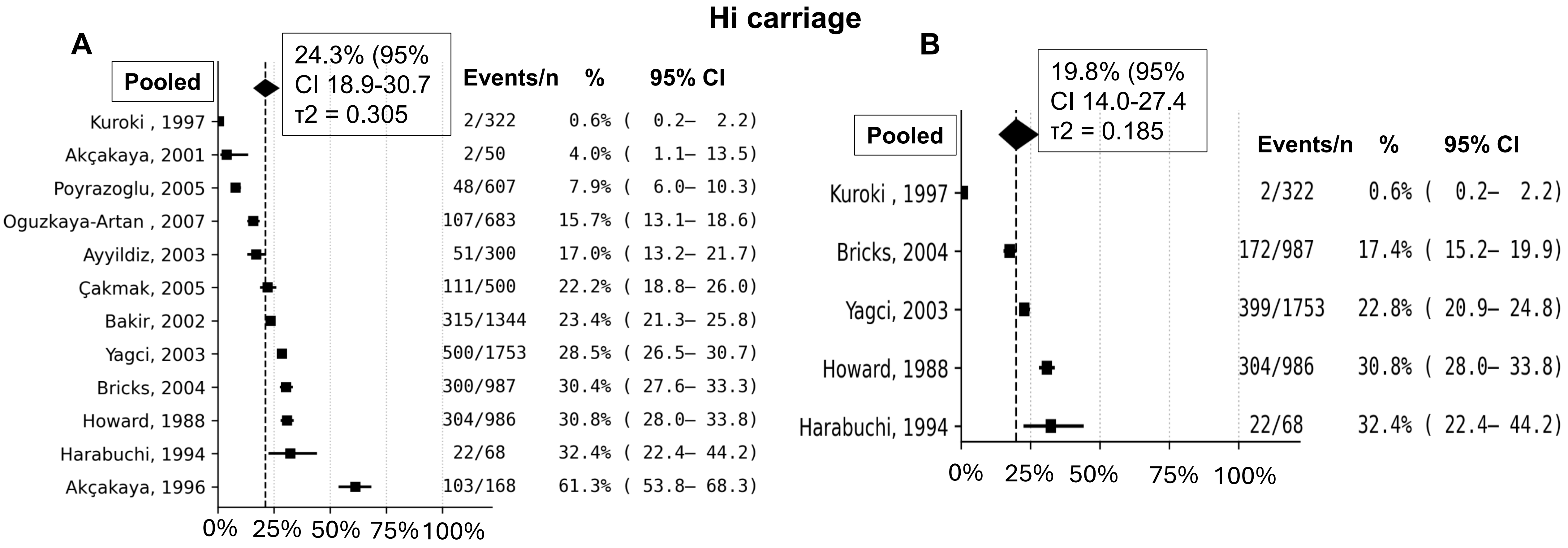

Supplementary Figure 1. Forest plot describing the carriage rate of Hi isolates before Hib vaccination. (**A**)Pooled estimations are shown within boxes. For each study Events/n indicates the number of carrier (Events) on the total number of subjects (n). The carriage percentage per study and it 95% CI are also shown. (B) A sensitivity analysis after excluding studies from Turkey.
