# Supplementary material for: Carriage of Haemophilus influenzae in the Pre- and Post-Hib Vaccine Eras Revisited: A Systematic Review and Meta-Analysis
Source: Vaccines (Basel). 2026 Jun 20;14(6):542. doi: 10.3390/vaccines14060542 (PMC13308107; doi:10.3390/vaccines14060542)
Supplement: Supplementary file 1 [file vaccines-14-00542-s001.zip › Supplementary Figure S2.pdf]

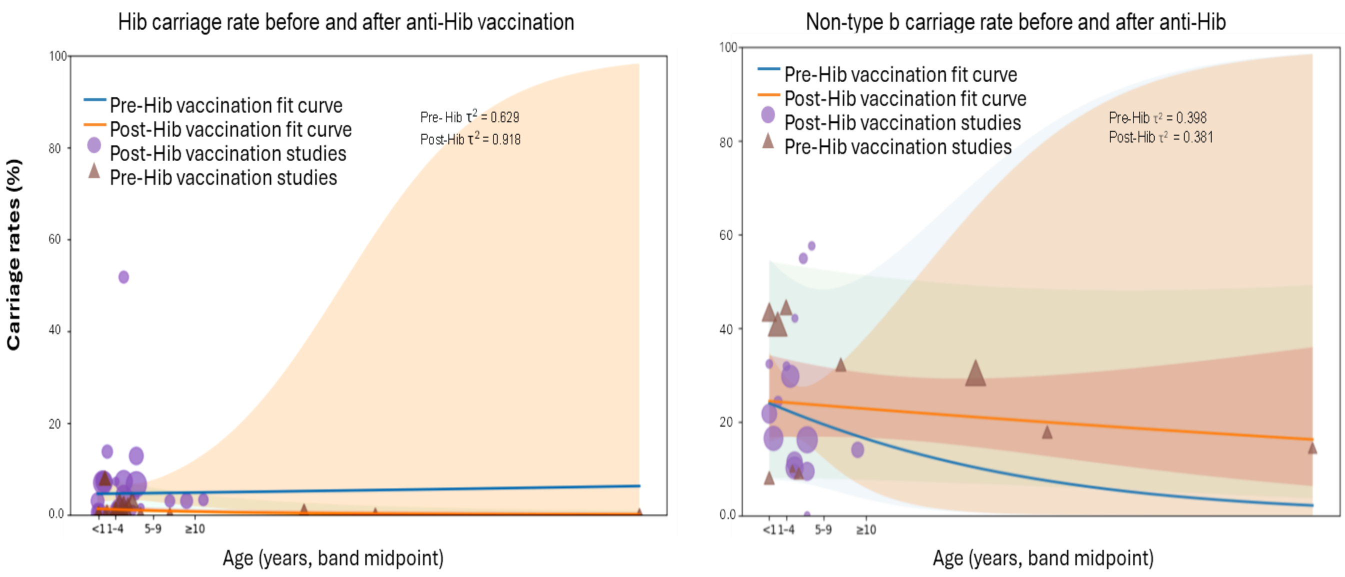

Supplementary Figure 2. Age–versus carriage meta-regression analyses. Hib and non–type b carriage rates were considered for the periods before and after Hib vaccination. Lines show random-effects meta-regression fits (logit scale) using age-band midpoints. Darker shading indicates 95% CI for the mean fit; lighter shading indicates 95% prediction interval. Circles and triangles represent the individual studies in both periods, and their size is proportional to N. τ^2 values are shown
