# Supplementary material for: Carriage of Haemophilus influenzae in the Pre- and Post-Hib Vaccine Eras Revisited: A Systematic Review and Meta-Analysis
Source: Vaccines (Basel). 2026 Jun 20;14(6):542. doi: 10.3390/vaccines14060542 (PMC13308107; doi:10.3390/vaccines14060542)
Supplement: Supplementary file 1 [file vaccines-14-00542-s001.zip › Supplementary Figure S3.pdf]

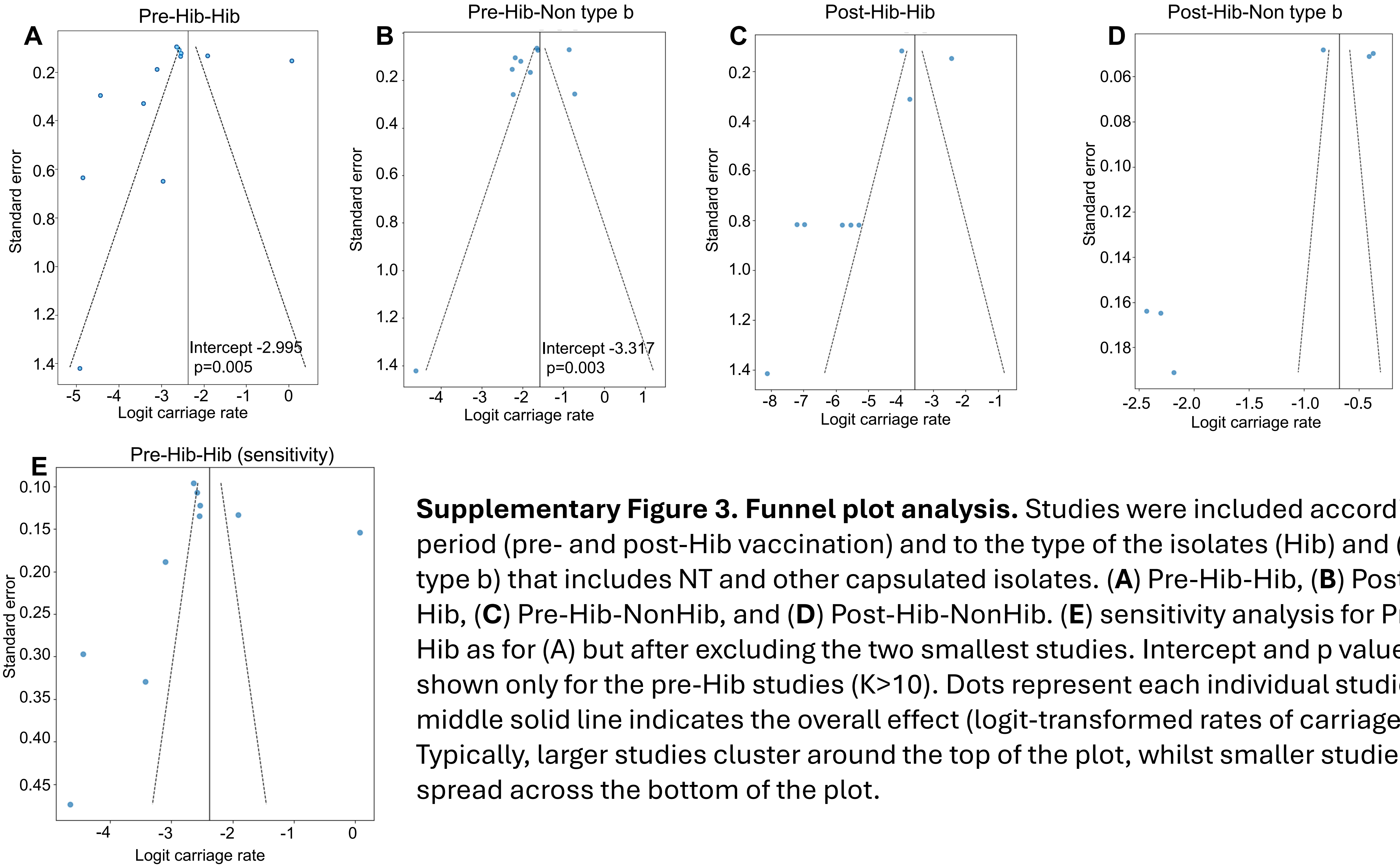

Supplementary Figure 3. Funnel plot analysis. Studies were included according to the period (pre- and post-Hib vaccination) and to the type of the isolates (Hib) and (Non–type b) that includes NT and other capsulated isolates. (**A**) Pre-Hib-Hib, (**B**) Post-Hib-Hib, (**C**) Pre-Hib-NonHib, and (**D**) Post-Hib-NonHib. (**E**) sensitivity analysis for Pre-Hib-Hib as for (A) but after excluding the two smallest studies. Intercept and p values are shown only for the pre-Hib studies (K>10). Dots represent each individual studies. The middle solid line indicates the overall effect (logit-transformed rates of carriage). Typically, larger studies cluster around the top of the plot, whilst smaller studies are spread across the bottom of the plot.
