# Supplementary material for: Carriage of Haemophilus influenzae in the Pre- and Post-Hib Vaccine Eras Revisited: A Systematic Review and Meta-Analysis
Source: Vaccines (Basel). 2026 Jun 20;14(6):542. doi: 10.3390/vaccines14060542 (PMC13308107; doi:10.3390/vaccines14060542)
Supplement: Supplementary file 1 [file vaccines-14-00542-s001.zip › Supplementary Table S1.pdf]

Supplementary Table 1. Included studies and their characteristics

Study, year	Country	Hib era	Age group (reported)	Age subgroups (if reported)	N sampled (denominator)	n carriers: all Hi	% carriers: all Hi	n carriers: Hib	% carriers: Hib	n carriers: other Hi (incl. NTHi/non-b)	% carriers: other Hi	Study setting	Study design
Harabuchi, 1994	USA	Pre-Hib	0-12 Months		68	22	32.40	0	0.00	22	32.40	Community-based pediatric primary care clinic in Buffalo, New York, USA.	Prospective cohort study of breast-fed infants followed from birth to 12 months with repeated nasopharyngeal cultures.
Yagci, 2003	Turkey	Pre-Hib	0-10 Years		1753	399	22.76	116	6.62	283	16.14	Respiratory clinic/hospital surveillance	Surveillance study
Howard, 1988	UK	Pre-Hib	0-72 Months		986	304	30.83	11	1.12	293	29.72	Community-based survey	Cross-sectional
		Pre-Hib		0-12 Months	585	130	22.22	3	0.51	127	21.71		
		Pre-Hib		13-24 Months	107	26	24.30	0	0.00	26	24.30		
		Pre-Hib		25-36 Months	69	27	39.13	5	7.25	22	31.88		
		Pre-Hib		37-48 Months	57	25	43.86	1	1.75	24	42.11		
		Pre-Hib		49-60 Months	102	57	55.88	1	0.98	56	54.90		
		Pre-Hib		61-72 Months	66	39	59.09	1	1.52	38	57.58		
Poyrazoglu, 2005	Turkey	Pre-Hib	3-24Months		818			59	0.07			Community-based	Cross-sectional
		Pre-Hib		3-6 Motnhs	400			12	0.03				
		Pre-Hib		7-12 Months	207			16	0.08				
		Pre-Hib		13-24 Months	211			29	0.14				
Akçakaya, 2001	Turkey	Pre-Hib	3-6 Years		50	2	4.00	2	4.00	0	0.00	Hospital/community pediatric population	Cross-sectional
Bakir, 2002	Turkey	Pre-Hib	< 2 years		1344	315	23.44	94	6.99	221	16.44	Community-based	Cross-sectional

Bricks, 2004	Brazil	Pre-Hib	<6 Years		987	172	17.43	72	7.29	100	10.13	Day-care centers	Cross-sectional
Ayyildiz, 2003	Turkey	Pre-Hib	7-12 Years		300	51	17.00	9	3.00	42	14.00	Community-based/pediatric population	Cross-sectional
Oguzkaya-Artan , 2007	Turkey	Pre-Hib	<7 Years		683	107	15.67	29	4.25	78	11.42	Community-based/preschool population	Cross-sectional
Akçakaya , 1996	Turkey	Pre-Hib	2-5 Years		168	103	61.31	87	51.79	16	9.52	Day-care centers	Cross-sectional
Kuroki , 1997	Japan	Pre-Hib	tnhs+ 9 years +13 Years		474			4	0.84			Community-based/pediatric population	Cross-sectional
		Pre-Hib		1-12 Months	322			2	0.62				
		Pre-Hib		13-48 Motnhs	152			2	1.32				
		Pre-Hib		9 Years	167			5	2.99				
		Pre-Hib		13 Years	154			5	3.25				
Çakmak, 2005	Turkey	Pre-Hib	3-6 Years		500	111		64	12.80	47	0.09	Community/pediatric population	Cross-sectional
Hammitt, 2014 and Hammitt, 2016	Kenya	Post-Hib	0-50 Years		2031	620	30.53	1	0.05	619	30.48	Population-based carriage surveys	Repeated cross-sectional surveys
		Post-Hib		<5 Years	623	278	44.62	1	0.16	277	44.46		
		Post-Hib		5-17 Years	509	164	32.22	0	0.00	164	32.22		
		Post-Hib		18-49 Years	483	86	17.81	0	0.00	86	17.81		
		Post-Hib		50 Years and olde	416	60	14.42	0	0.00	60	14.42		
McVernon, 2004	UK	Post-Hib	1-4.99 Years		3936			72	1.83			Community carriage survey	Cross-sectional
		Post-Hib		1-1.99 Years	117			0	0.00				
		Post-Hib		2-2.99 Years	853			9	1.06				
		Post-Hib		3-3.99 Years	2155			37	1.72				
		Post-Hib		4-4.99 Years	811			26	3.21				
Abu Sailik, 2020	Jordan	Post-Hib	0.5-6 Years		300	31	10.33	1	0.33	30	10.00	Hospital/community pediatric population	Cross-sectional
Hu, 2016	China	Post-Hib	1-1.5 Years		614			49	7.98			Community vaccination clinics	Cross-sectional
		Post-Hib		1 Year	170			14	8.24				
		Post-Hib		1.01-1.5 Years	444			35	7.88				

Millar, 2000	USA	Post-Hib	<7 Years		381			1	0.26			Community-based	Cross-sectional surveillance
Gönüllü, 2020	Turkey	Post-Hib	1-7 Years		445	50	11.24	10	2.25	40	8.99	Hospital/community pediatric population	Cross-sectional
Shirvani, 2019	Iran	Post-Hib	<1 year		500	41	8.20	1	0.20	40	8.00	Health-care centers	Cross-sectional
Romanin, 2007	Argentina	Post-Hib	1 and 5 years		1600	641	40.06	1	0.06	640	40.00	Community-based surveillance	Cross-sectional
		Post-Hib		1 year	900	392	43.56	1	0.11	391	43.44		
		Post-Hib		5 Years	700	249	35.57	0	0.00	249	35.57		
Dabernat, 2003	France	Post-Hib	<3 Years		1683	688	40.88	0	0.00	688	40.88	Day-care centers	Cross-sectional molecular epidemiology study
